# Paediatric pineal region cysts: enigma or impaired neurofluid system?

**DOI:** 10.1007/s00381-023-06000-4

**Published:** 2023-06-01

**Authors:** Hans C. Ludwig, Steffi Dreha-Kulaczewski, Hans Christoph Bock

**Affiliations:** 1 Department of Neurosurgery, Division of Pediatric Neurosurgery, University Medical Center Göttingen, Robert-Koch-Str. 40, 37075 Göttingen, Germany; 2 Department of Pediatrics and Adolescent Medicine, Division of Pediatric Neurology, University Medical Center Göttingen, Robert-Koch-Str. 40, 37075 Göttingen, Germany

**Keywords:** Pineal cyst, Hydrocephalus, Microsurgery, Real-time MRI, Respiration, Glymphatic system

## Abstract

**Purpose:**

Pineal region cysts (PCs) may affect the tectum and aqueduct and cause deep central vein congestion. Beside headaches, PC often causes a broad range of symptoms, leading to prolonged diagnosis and therapy. The aims of this study are to reveal parameters that might explain the ambiguity of the symptoms and to identify factors in association with the respiration-driven neurofluid system.

**Methods:**

This retrospective study included 28 paediatric patients (mean age 11.6 years) who received surgical treatment and 18 patients (mean age 11.3 years) who were followed conservatively. Symptoms, time to diagnosis, cyst size, ventricular indices, head circumference and postoperative outcome, were analysed. Four patients were investigated for CSF dynamics with real-time MRI. The mean follow-up time was 1.6 years.

**Results:**

The most common early onset symptoms were headaches (92%), blurred vision (42.8%), sleep disturbances (39.3%) and vertigo (32.1%). Tectum contact was observed in 82% of patients, and MRI examinations revealed that imaging flow void signals were absent in 32.1% of patients. The maximal cyst diameters were 13.7 × 15.6 mm (mean). Together with a postoperative flow void signal, 4 patients recovered their respiration-driven CSF aqueductal upward flow, which was not detectable preoperatively. After surgery the main symptoms improved.

**Conclusion:**

Despite proximity to the aqueduct with frequently absent flow void signals, hydrocephalus was never detected. Data from real-time MRI depicted a reduced preoperative filling of the ventricular CSF compartments, indicating a diminished fluid preload, which recovered postoperatively.

## Introduction

Pineal cysts (PC) can often be completely asymptomatic and present as incidental findings in children and young adults, with a prevalence of 0.6–23% in the general population and a prevalence of up to 40% in autopsies [1]. Sometimes, neurosurgeons see patients with a wide range of complaints and a pineal cyst on MRI, which makes decisions about surgical or conservative therapy difficult [2, 3]. Some reports, studies and meta-analyses have examined PC; however, the data on paediatric patients are scarce. Chocque-Velaszquez et al. [4] examined 109 paediatric patients from 43 study records (see Table 1 for an overview).

**Table 1:** Overview of the literature on PC in adults and children [2-13]

**Author**	**Year**	**Period (years)**	***N***	**Centres**	**Age**	**Children**	**f > m**
Uschold	2011	n.n	6	1	26	2	y
Kalani	2015	14	18	1	24	Few	y
Majovski	2017	16	110	1	n.n	No	n.n
Majovski	2017	n.n	4	1	n.n	No	n.n
Choque-Velasquez	2019	18	60	1	29	n.n	y
Koziarski	2019	n.n	28	1	31	Few	y
Pitskhelani	2019	21	25	1	> 18	No	y
El Damaty	2019	15	43	3	25, 6	Few	y
Milton	2020	n.n	24	11	50	Few	y
Yeung	2021	n.n	97	1	n.n	n.n	y
Mendoza	2021	n.n	84	1	n.n	No	y
Jenkinson	2021	Review		1		No	y

*n.n. *Not known

Altogether, there are few reports in the literature regarding PCs among children, and consensus guidelines for PC management or treatment are still not available. PC derived from the pineal parenchyma cannot be easily distinguished from arachnoid cysts of the pineal region cisterns and 3rd ventricle. In contrast to other tumours requiring volume in the pineal region [14, 15], PCs are rarely accompanied by hydrocephalus despite aqueductal stenosis [11]. Some researchers have attributed PCs to elevated venous pressure [16] and elevated CSF pulsatility [17]. Aqueductal stenosis is expected to enlarge the supratentorial ventricular volumes. Symptomatic PCs seem to offer further understanding of related fluid dynamics if we scrutinise current concepts on hydrocephalus pathophysiology [18-23]. It has become obvious that the traditional concept established by Dandy has been turned towards novel, more complex roles of the aqueduct. In particular, the finding of the centripetal ventricular fluid filling by inspiration has lead to the current controversy of the classical concepts. Along this line, in early studies, Klarica et al. [24] could not demonstrate elevation of ICP during 2 h of measurements in a cat model following iatrogenic aqueductal stenosis. We have recently published experiences from ETV procedures in which we showed CSF filling upward through the opened stoma [25, 26]. Several clues have been identified regarding the concept of a strong regulatory role for the aqueduct with a small radial diameter, whose value accounts for the 4th power according to the law of Hagen-Poiseuille. This means that any small deviation of its diameter induces laminar fluid flow alterations in the fourth power. Because no alternative pathway exists for fluid movement, inspiration-induced CSF flow can exclusively enter the ventricles through the aqueduct, thereby functioning as a control valve to adapt to body positions and activity. Because of the transtentorial connection of the quadrigeminal cistern to the 3rd ventricle, intracranial or intraventricular pressure can be applied to the aqueduct for adaptation purposes. This mechanism allows pressure adaptations by the aqueductal diameter and a sort of fine tuning [25].

If such a mechanism exists, any adjacent cyst volume at the surrounding aqueductal environment should lead to the deterioration of the valve function like an added offset value in a regulatory pathway.

Therefore, the current single-centre study aimed to examine paediatric patients with fenestrated and unfenestrated PCs to identify different exploratory variables that might further elucidate the pathophysiology. In addition to clinical data, we concentrated on the symptomatology, duration of symptoms before diagnosis, geometry of the cysts, and ventricular sizes. In a small group of patients, we performed additional imaging (e.g. real-time flow MRI) to analyse the CSF-flow dynamics.

## Patients and methods

The retrospective study was conducted from 2007 to 2021 in a university hospital centre using prospectively collected data from our institutional paediatric neurosurgical patient registry [27].

Most patients were referred directly for surgical consideration. Four patients with isolated headaches and sleep disorders without further symptoms were treated ex iuvantibus with 2–4 mg melatonin at night. Two of them experienced sustainable headache relief, and the other two patients with persistent headaches were treated by surgery. Decision making for surgery was dependent on isolated headaches (6) or together with vision (3) and sleep disorder (7), vertigo (5), vomiting (3) and dizziness (6). In some cases (2), differentiating between CSF-related symptoms and other causes of headache was not possible. The decision to proceed with surgery in those cases consisted on cyst size, presence of aqueduct compression on imaging, and obstinacy of symptoms. Patients who received microneurosurgical cyst fenestration via a suboccipital midline craniotomy underwent operations in a “sitting prayer position”, as described by Choque-Velasquez et al. [28]. The head was fixed in a Mayfield clamp, and patients were equipped with a centrally positioned venous line and transoesophageal ultrasound probe to detect air bubbles in case of aspiration. Postoperative ICU care was performed overnight, and the mean hospital admission time was 9 days. Postoperative control was performed after 4 h by cCT. Each patient underwent postoperative MRIs at 3-month intervals. Four patients were investigated further pre- and postoperatively via real-time flow MRI [21, 22]. Patients without specific complaints and surgical therapy did receive follow-up in our joint outpatient ambulance by paediatric neurologists and consulting paediatric neurosurgeons. Statistical calculations and graphics were performed with Statistica™ (TIBCO Software Inc. Palo Alto, CA 94304, USA) using ANOVA. Informed consent was obtained from patients or their caregivers. The institutional review board of the Georg August-University Goettingen approved the study (12–9-17), and the study complied with the Declaration of Helsinki.

### Real-time phase-contrast flow MRI

All datasets were acquired on a 3 Tesla scanner (Magnetom Prisma Fit, Siemens Healthcare) using real-time phase-contrast flow MRI based on highly undersampled radial FLASH sequences as described [20-22, 29, 30]. Measurements in sagittal or coronal orientation to the stoma in the floor of the 3rd ventricle were conducted with a 64-channel head coil.

### RT-MRI data analysis

Real-time flow MRI datasets were quantitatively analysed using CaFuR software (Fraunhofer Mevis, Bremen, Germany) [31] designed to accomplish automatic segmentation of flow signals in real-time image series. Manual definition of an initial ROI at the level of the aqueduct for the determination of through-plane flow was based on both signal intensities in magnitude images and corresponding phase difference values in velocity maps. Further data processing was performed using MATLAB (Mathworks, MA, USA), including net flow calculation and data visualisation.

## Results

**Fig. 1:**
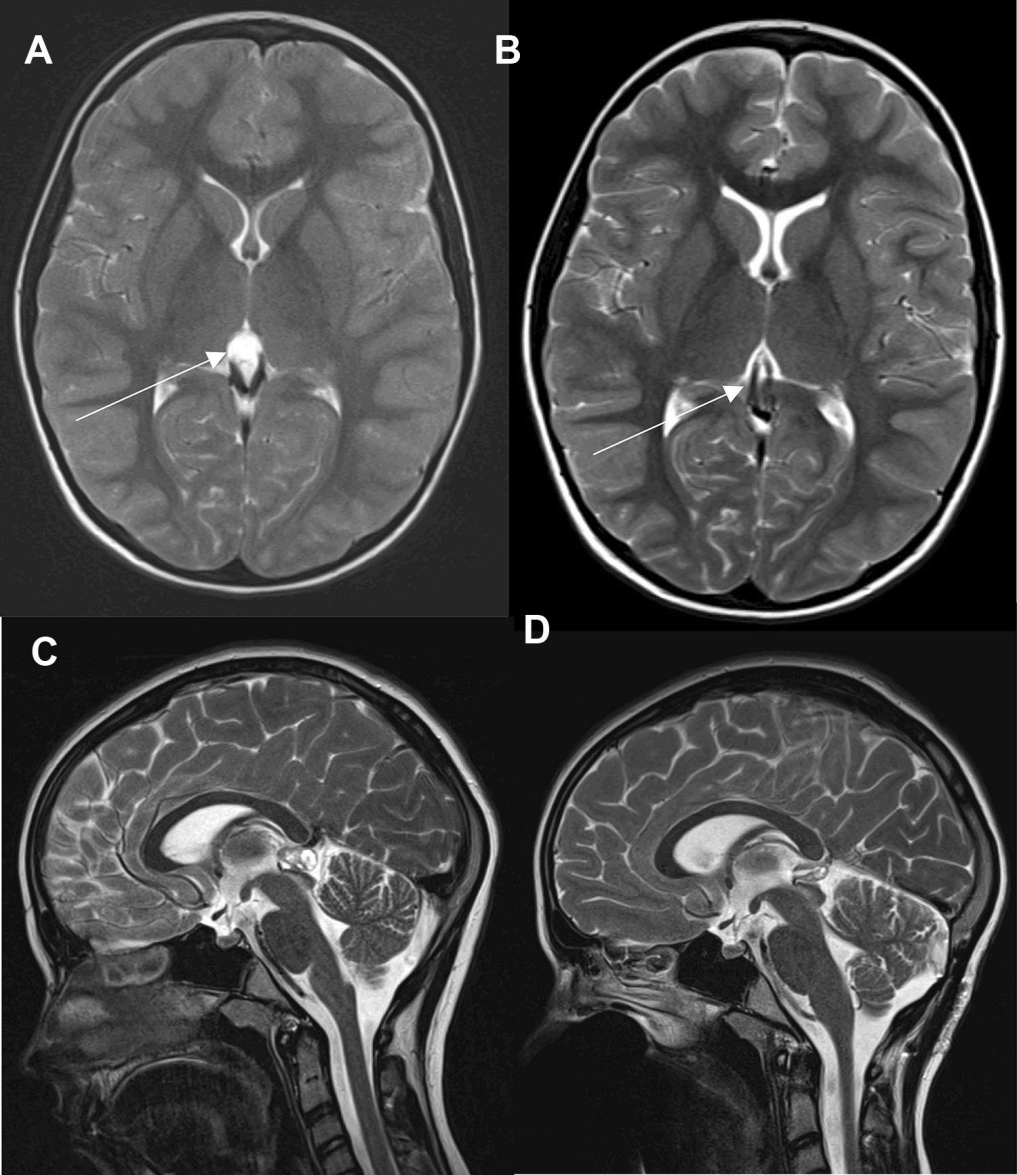
Typical aspect of a symptomatic multisegmented pineal cyst with tectum contact, venous congestion (arrow), small ventricular diameter, and a cyst diameter of more than 10 mm preop (**A**). Postop after 4 months slight alteration of the ventricular size, uncongested veins (arrow) (**B**). Pre- and postoperatively sagittal view with missing pre- (**C**) and patent flow signals (**D**) after microsurgical fenestration. Loculated parenchymal pineal cyst preoperatively and reduced size postoperatively within the pineal body

The surgically treated children (*N* = 28) were between 10 months and 17 years old, the mean age was 11.6 years, and the female/male ratio was 1.15. The mean time interval from diagnosis to operation was 11.8 months, with a follow-up interval of 1.3 years. Nonoperated children (*N* = 18) were 11.3 years old, with a female/male ratio of 3.5. For this group, the follow-up interval was shorter, with a mean of 1.02 years. The interval between symptoms and first admission was 11.6 (11.3) months, reaching a maximum of 5 years. BMI was similar in both groups, with 20.21 and 23.1, and the mean head circumference was 66 P (63 P for nonoperated children). Symptoms leading to admission included headaches (92%), blurred vision (42.8%), sleep disturbances (39.3%) and vertigo (32.1%). Two patients had a papilledema preop. Direct contact of the PC to the tectum was counted in 82%, and a missing flow void signal was found in 32.1% (Fig. 1).

The maximal cyst diameters were 13.7 mm for the axial axis and 15.6 mm (mean) for the longitudinal sagittal axis. For nonoperated patients, cyst diameters and lengths were estimated as 8.1 × 9.1 mm, each below 1 cm. Ventricular sizes were never hydrocephalic, instead “normal” or even classified “small” with mean Evans ratio (0.26) and FOHR (0.33). Physical strain during sportive activity (25%) and educational schooling disorders were estimated as 21%. Together with a postoperative flow void signal in 64.3%, 4 patients re-established their respiration-driven CSF upward flow by real-time MRI, which was not detectable before surgery (Fig. 1). The mean ER (0.25) and FOHR (0.33) were not significantly altered at postoperation. Indicating communication, the occurrence of air inside the ventricles 4 h after the operation was 75% (Table 2 for details). Among the 4 patients treated with melatonin, 2 (2 mg/night) had complete headache relief.

**Table 2:** Descriptive statistics

**Variable**	**OP**		**Non-OP**		
	***N***	**%**	***N***	**%**	***p*** **value**
**Patients**	28		18		
f/m	1.15		3.5		0.10
Age OP (mean)	11.68		-		-
Age (years) at time of decision (mean)	11.68		11.3		0.38
Height (cm) (mean)	160.3		149.7		0.20
Weight (kg) (mean)	47.66		60.8		
BMI (mean)	20.21		23.1		0.14
FU (years) (mean)	1.37		1.02		-
Interval (mean)	11.8		7.8		0.48
Progression of complaints	3	10.70	0	0.00	0.18
Head circumference (mean P)	66		70		0.95
**MRI presentation**					
Cyst diameter (mm) (mean)	13.7		8.1		0.01
Cyst length (mm) (mean)	15.6		9.1		0.01
Contrast enhancement of cyst wall	2	0.07	0	0.00	0.26
Multiple compartments	4	14.20	4	22.22	0.50
Tectum contact	23	82.10	15	83.33	0.01
Aqueductal Flow void pre	9	32.00	12	66.67	0.21
ER pre (mean)	0.26		0.23		0.04
FOHR pre (mean)	0.33		0.31		0.79
**Complaints/symptoms**					
Headache	26	92.8	12	66.67	0.07
Nausea/vomiting	3	10.70	5	27.78	0.63
Vertigo	9	32.00	4	22.22	0.72
Vision	12	42.8	5	27.78	0.20
Dizziness	6	21.00	4	22.22	0.78
Sleep disorder	11	39.2	9	50.00	0.74
Sport disorder	6	21.40	1	5.56	0.13
Education disorder	7	25.00	3	16.67	0.29
Seizures	3	10.70	0	0.00	0.17
**Treatment and outcome**					
Melatonin treatment	0		4	22.00	-
Flow void postoperation (po)	18	64.30	-	-	-
ER po (mean)	0.25	-	-	-	-
FOHR po (Mean)	0.33	-	-	-	-
Headache po	5	7.10	-	-	-
Vomiting po	0	-	-	-	-
Vertigo po	0	-	-	-	-
Vision disorder po	2	7.10	-	-	-
Sleep disorder po	0	-	-	-	-
Air inside ventricles at 4 h po	21	75.00	-	-	-

*ER* Evans ratio, *FOHR* Fronto occipital horn ratio, *po* Postoperation

In nearly all cases (93%), the leading symptoms improved or remained stable after the operation.

**Fig. 2:**
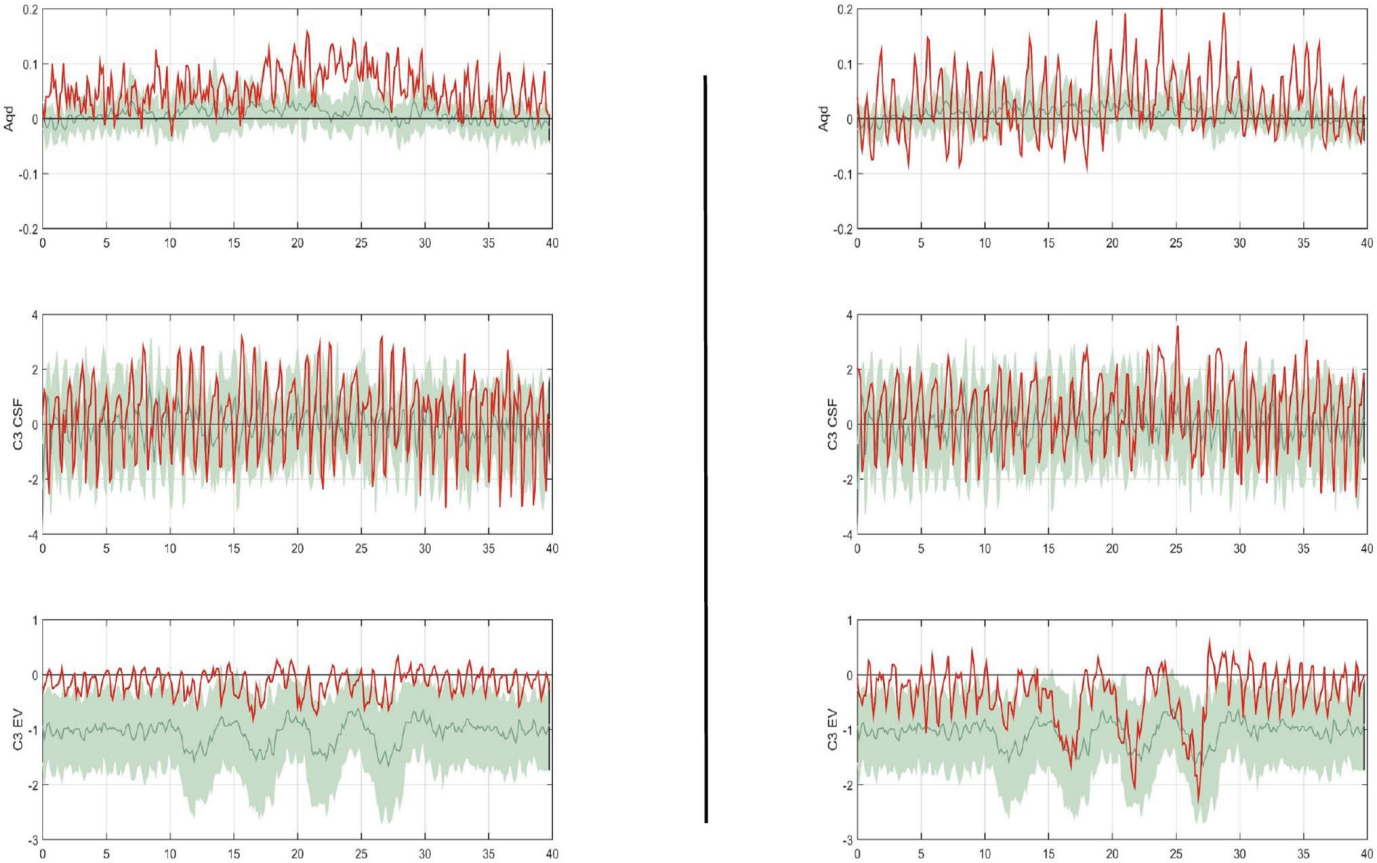
RT-MRI data showing one individual (red line) with (left) preoperative periods of normal breathing followed by deep inspiration after 10 s with consecutively altered venous blood dynamics at the level of C3 and CSF dynamics at level C3 and measured at the aqueduct (Aqd) for 20 s. On the right side, equal parameters at level C3 but higher CSF flow through the aqueduct. The underlying green-coloured mantle curves (mean and amplitude) are an individual overlay on data from a healthy study group for comparison [19]

Nine patients showed a patent flow void inside the aqueduct on the postoperative MRI, in which no flow void could be distinguished preoperatively. There was no mortality or morbidity. The mean length of hospital stay was 9.4 days. The real-time MRI data showed significantly higher CSF upward flow inside the aqueduct at postoperation than at preoperation (Fig. 2).

**Fig. 3:**
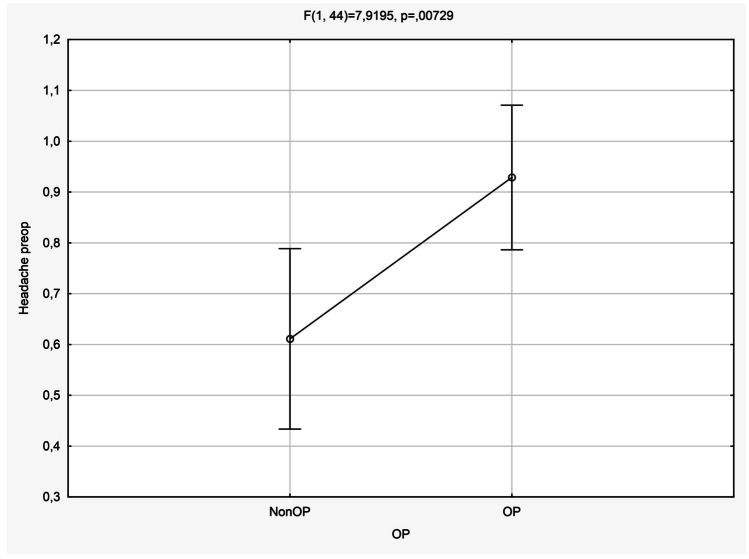
Headache as a significant (*p* = 0.007) main symptom in patients with and without microsurgical fenestration

**Fig. 4:**
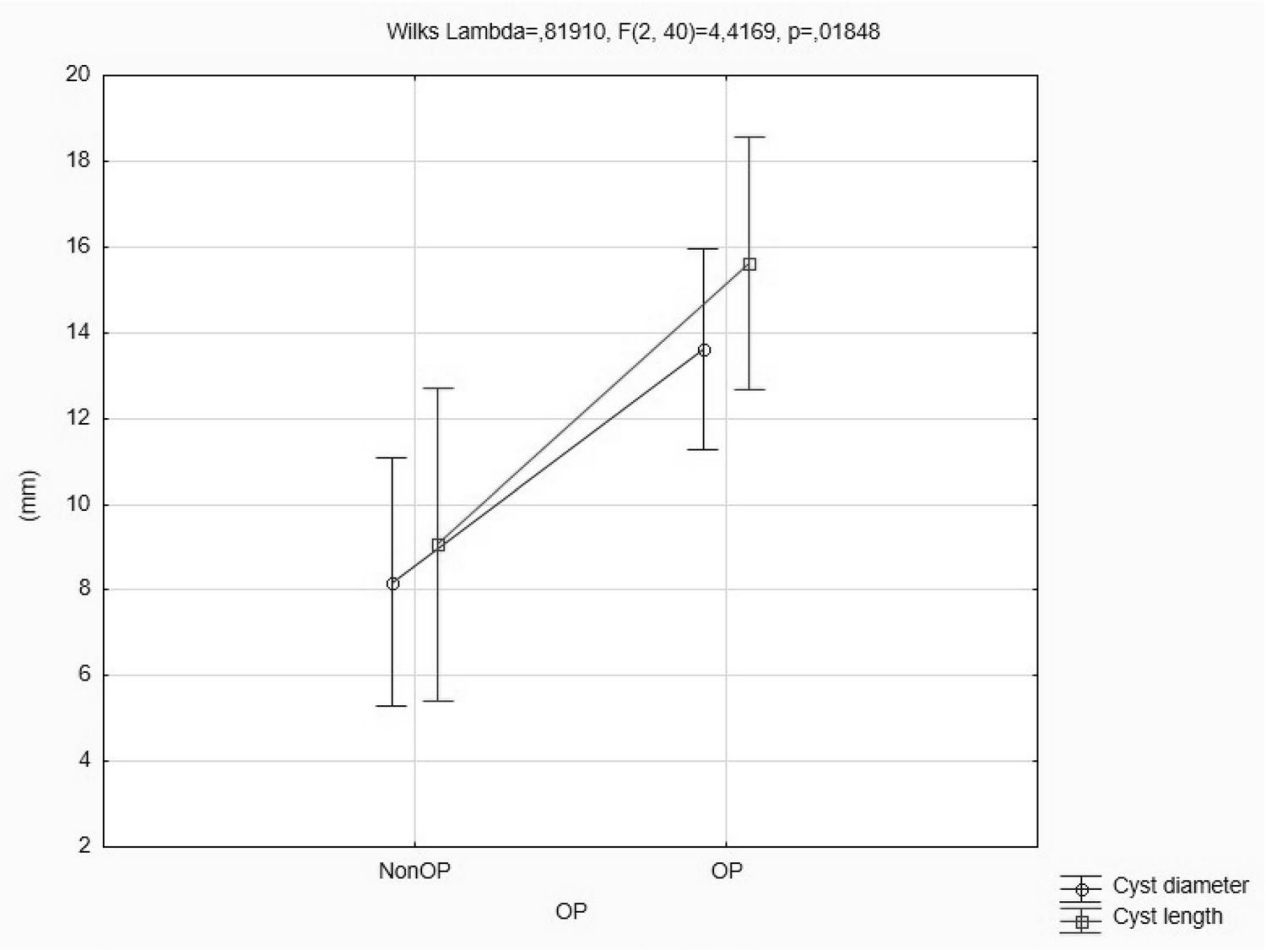
Diameter and sagittal length for operated and nonoperated cysts (*p* = 0.018)

**Fig. 5:**
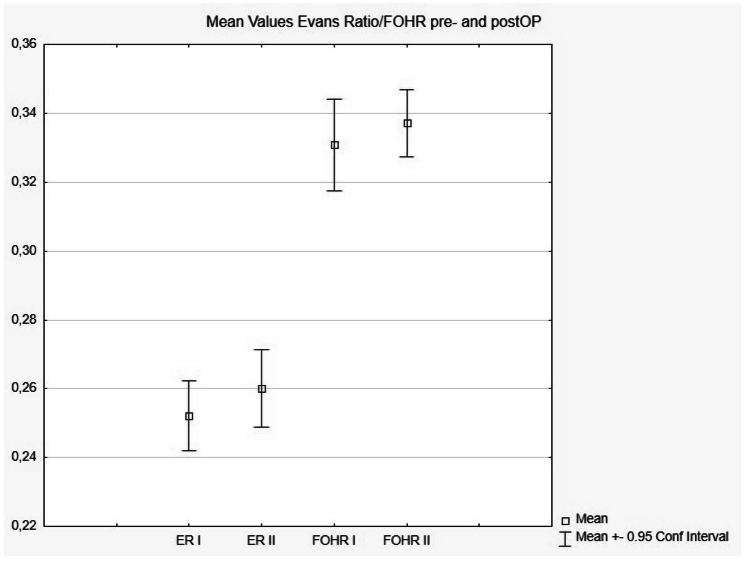
Evans’s ratio (ER) and FOHR for patients who received microsurgical fenestration

Statistical calculations demonstrated the leading symptom of headaches (Fig. 3) as the only significant parameter for each group of operated and non-operated children (*p* = 0.07). MRI parameters were significant for cyst diameter and length (*p* = 0.01), tectum contact of the cyst (*p* = 0.01) and Evan’s ratio preoperatively (*p* = 0.04) (see Figs. 3, 4 and 5; Table 2).

## Discussion

PC occur in children and adults [32]. They are considered to be of benign character without any symptomatology and without the need for further follow-up.

They are detected incidentally by MRI in children at a rate of 57% with a mean diameter of not more than 6 mm, and they are often multicystic parenchymal cysts without thickened walls or contrast enhancement [33]. Usually, females are more likely to be affected than men, with a female/male ratio of 1.5. For several years, a growing number of studies have described (Table 1) PC disease, which might lead to headache, nausea and vomiting, dizziness and vertigo, sleep disturbances with deferred sleep induction and daytime sleepiness. Some patients report blurred vision, and in childhood, some patients develop educational difficulties with problems at school and aversion to physical activity. Most patients lack clinical signs of hydrocephalus. Differentiation from other types of headaches, which belong to the main burden of diseases in school-aged children and young adolescents, is rather difficult [34]. Melatonin deficiency has been identified by some authors as a reason for symptomatology of PCs [7], but the results were inconsistent. Usually, clinical management of these patients is carried out by neurologists, and in the case of MRI imaging, radiologists and neurologists deny any further indication with regard to the usually small ventricular dimensions and the missing Parineaud syndrome. This diagnostic path may be the main reason for the long diagnostic delay after the beginning of complaints. If those patients are presented occasionally to the neurosurgeon, some of the reported symptoms resemble those of patients with arachnoid cysts or idiopathic intracranial hypertension (IIH). Indeed, the leading symptoms are severe and frequent headaches. Getting aware of this symptomatology, a growing number of neurosurgeons tend towards microsurgical fenestration, which often and statistically significantly resolves or at least weakens complaints [11, 12]. The indication for surgery should consider reported cases with severe surgical morbidity and even mortality [4] despite higher grades of surgical experience. A common feature for the broad symptomatology is the lack of any hydrocephalus and Parineaud syndrome, which belong to the main classic cornerstones of space-occupying lesions of the pineal region. Given the anatomical specialties of this region, four main pathophysiological systems seem closely related. (1) The close vicinity to the aqueduct interferes with the CSF passage. (2) The close vicinity to the central veins could challenge venous drainage. (3) Contact with the quadrigeminal plate could affect visual accommodation, hearing sensations and proprioception. (4) Finally, the endocrine function for secretion and distribution of melatonin could be affected. In contrast to the pineal gland, any cystic alteration of the pituitary gland of similar size would be expected to cause severe symptomatology. We cannot explain why any cystic space-occupying process inside the pineal region with densely packed content and densely sealed arachnoid membranes (Fig. 3) of appropriate size contacting the deep veins and the tectal plate is not able to cause aqueductal stenosis with elevated mean ICP and hydrocephalus. In such investigations, only ICP pulsatility scores were elevated [17] but not ICP mean values themselves. Eide and Ringstad reported shunt therapy in some cases with PC and addressed a higher central venous pressure as a main cause of the disease [16]. We therefore searched for parameters in our patient cohort with pineal cysts treated surgically or followed conservatively, which could further explain the specificities of the disease. Decision-making in such cases, as long as no clear evidence exists, is partly led by the clinical experiences of the surgeons. To enhance the insights into the underlying pathophysiology, we have tried to include our experience with CSF flow [23]. In contrast to traditional concepts, sustainable CSF upward flow caused by inspiration is the most important part of the fluid exchange system of CSF with interstitial fluid and its entrance into the glymphatic system, which was traditionally called the “minor pathway” [23, 35, 36]. Sustainable flow is caused by deep inspiration only during non-REM sleep [37], whereas cardiac-triggered oscillations cause fast but ineffective fluid movements that are visible on conventional MRI scanners or cine MRI as flow void signals. Conventional phase contrast MRI techniques inherently cancel breathing component of the CSF dynamics [38]. This has been outlined recently in detail by our group [39]. An important part of this fluid flow passes the aqueduct in the upward direction. The aqueduct is the unique fluid conducting element with a small diameter and a characteristic curve following a mathematical formula, in which the radius is calculated by the 4th power according to Hagen and Poiseuille’s statute. This tuning element is severely disturbed in aqueductal stenosis, for example, by membranes or tumours. Whereas pure stenosis always leaves a tiny residual hole for at least some fluid passage, in the case of tumour-related complete stenosis, ventricular autonomy can be reached without any ventricular enlargement. Hydrocephalic triventricular dilatation, on the other hand, appears to be the result of incomplete stenosis due to a CSF trapping mechanism [25]. If a similar mechanism is the underlying cause of a cystic space-occupying process with contact to the tectal plate and aqueductal flow hindrance, the equilibrated system should deteriorate with regard to its regulating function. We have therefore emphasised in our study those parameters, which might agree with this hypothesis in a cohort of 28 surgically treated children compared to the second cohort, in which the decision for conservative treatment was favoured. Similar ages, similar female/male ratios, body weight and height, BMI, ventricular indices ER and FOHR and similar symptoms were analysed. The main significant differences were the severity and frequency of headache, cyst diameter and detectable aqueductal flow void signal in T2-weighted images. In accordance with the recent literature, nearly all reported patients did not suffer from hydrocephalus, but headaches, blurred vision nausea and disturbed sleep induction were the main symptoms [10]. After surgical treatment, in more than 90% of the patients, the symptoms disappeared. In our paediatric patient population, we observed the same trend. The most convincing details were the significant difference regarding the frequencies of headaches, the size of the cyst and the ventricular dimensions in those patients who received surgery compared to the children who received conservative therapy. We did not expect any certain or significant increase in ventricular size after the surgery. The visible widening of the frontal horns in several MRIs postoperatively is below the statistical threshold of the ventricular indices (Fig. 5). Therefore, the results of the real-time MRI investigations are most important, as they could show a certain inspiration triggering CSF flow movement upwards through the postoperatively unhindered aqueduct. This upward stream is necessary for the preload of the interstitial fluid flow system and the termination of complaints. This has already been shown by our group for ETV in the treatment of obstructive hydrocephalus [19, 20] and confirms our hypothesis of a deregulated aqueductal function as the main cause of the disease.

## Conclusion

Our data of 28 children with microsurgical fenestration of PC compared to a cohort of 18 conservatively treated children could show striking differences with regard to the main symptom of headache and the significance of the cyst diameters. Only a few patients experienced headache relief with sleep induction by melatonin. After microsurgical fenestration of pineal cysts preserving the pineal gland, most children were free of headaches, nausea, vomiting, blurred vision or cognitive deficits. Part of the pathophysiology seems to be a disturbed fluid exchange system by any offset to the tuning features of the aqueduct, in which its radius is calculated by the 4th power according to Hagen-Poiseuille’s law. Therefore, most patients had a slight elevation of the postoperative ventricular indices, even if the data were not significant. Similar mechanisms of the disturbed neurofluid system are involved in obstructive hydrocephalus and might be directly involved in pseudotumor cerebri. Our experiences could lead to more differentiated decision-making in treating this PC disease.

## Data Availability

All data have been included into the tables.
